# Geosocial Networking App Use Associated With Sexual Risk Behavior and Pre-exposure Prophylaxis Use Among Gay, Bisexual, and Other Men Who Have Sex With Men: Cross-sectional Web-Based Survey

**DOI:** 10.2196/35548

**Published:** 2022-06-13

**Authors:** Laurel P Gibson, Emily B Kramer, Angela D Bryan

**Affiliations:** 1 Department of Psychology and Neuroscience University of Colorado Boulder Boulder, CO United States

**Keywords:** dating app, mobile dating, hookup, gay, pre-exposure prophylaxis, sexual risk, HIV, STI, mobile phone

## Abstract

**Background:**

In the United States, geosocial networking (GSN) apps (ie, mobile dating apps) have become central to dating and sexual interactions in recent years. Among gay, bisexual, and other men who have sex with men (GBM), these apps play an important role in reducing barriers and facilitating partner seeking. However, despite these benefits, there are concerns that these apps may facilitate risky sexual behavior and transmission of sexually transmitted infections (STIs) among GBM.

**Objective:**

This study aimed to examine the association between GSN app use and sexual risk in a US sample of GBM.

**Methods:**

Using a cross-sectional design, respondents (N=223) completed a web-based survey assessing their use of GSN apps, sexual risk and protective behaviors, HIV serostatus, and previous STI diagnoses.

**Results:**

Respondents were aged 21-78 (mean 31.90, SD 10.06) years and 69.5% (155/223) were non-Hispanic White. The sample included respondents from 40 states and the District of Columbia. Nearly half (104/223, 47%) of the participants reported using GSN apps. GSN users were more likely to report past-year condomless anal intercourse (*P*<.001), 3 or more sexual partners in the previous year (*P*<.001), and a previous STI diagnosis (*P*=.001) than nonusers. GSN users also reported more frequent use of recreational drugs before sex (*P*=.001), alcohol use before sex (*P*<.001), and cannabis use before sex (*P*=.01). Interestingly, GSN users were also more likely to report having ever taken an HIV test (*P*<.001) and using pre-exposure prophylaxis (*P*=.03). The rates of HIV seropositivity did not differ significantly between GSN users and nonusers (*P*=.53). Among the subset of GSN users, 38 participants reported using only GBM-specific GSN apps (eg, Grindr), whereas 27 participants reported using only sexuality nonspecific GSN apps (eg, Tinder). Exclusive users of GBM–specific apps reported more frequent recreational drug use before sex (*P*=.01) and were also more likely to report past-year condomless anal intercourse (*P*<.001), 3 or more sexual partners in the previous year (*P*=.004), a previous STI diagnosis (*P*=.002), and HIV testing (*P*=.003). Alcohol use before sex, cannabis use before sex, pre-exposure prophylaxis use, and HIV rates were similar between both groups (*P*>.11).

**Conclusions:**

The findings suggest that GSN apps may be a useful pathway for interventions aimed at reducing STI risk in GBM. Future prospective studies should examine how risk levels change after the initiation of GSN app use.

## Introduction

### Background

Although gay, bisexual, and other men who have sex with men (GBM) make up a small fraction of the US population, they are disproportionately affected by HIV, comprising nearly 70% of new HIV cases in the United States in 2018 [1]. Furthermore, GBM experience elevated rates of several sexually transmitted infections (STIs) beyond HIV, including syphilis, human papillomavirus, and hepatitis [2-5]. The disproportionate burden of STIs experienced by this community may be driven, in part, by higher rates of risky sexual behaviors, which are known to increase susceptibility to STIs, including HIV [6].

The US Centers for Disease Control and Prevention have identified GBM as a primary target group for HIV and STI prevention efforts, emphasizing the need for tailored interventions aimed at reducing risk behaviors among GBM. As such, there is a critical need to identify factors associated with sexual risk among GBM in order to inform our approach to mitigating the transmission of STIs, including HIV.

The use of geosocial networking (GSN) apps (ie, mobile dating apps) may be a key factor associated with sexual risk in GBM. GSN apps are smartphone apps that use GPS data to allow individuals seeking romantic or sexual partners to quickly connect and chat with other users nearby. The popularity of GSN apps (eg, Tinder and Grindr) has surged in recent years [7], most notably during the COVID-19 pandemic [8], and there is broad consensus that these apps have become central to dating and sexual interactions, particularly among GBM. According to recent survey data, 55% of lesbian, gay, and bisexual (LGB) adults, including GBM, have used a dating website or app at some point compared with 28% of their heterosexual counterparts [9]. The popularity of dating apps among LGB adults has been attributed to their role in addressing the barriers to meeting potential sexual partners typically faced by members of sexual minority groups, such as stigma and a desire for anonymity and discretion among those who prefer not to publicly identify as LGB [10].

Despite these benefits, there is also evidence supporting concerns that GSN apps may facilitate risky sexual behavior and transmission of STIs among GBM. Although it is possible that GSN apps may directly promote riskier behaviors, it is also possible that the innovative features of these apps that facilitate connections among LGB individuals attract individuals seeking to engage in risky behaviors (ie, self-selection) [11]. For instance, the use of GSN apps may reflect greater engagement in subcultures with more accepting norms toward sexual risk taking (eg, sexualized drug use) [12,13]. Regardless of the mechanism, the rates of STIs are high among app-using GBM [6], and a recent meta-analysis examining GSN app use among GBM identified a greater likelihood of STI (ie, syphilis, gonorrhea, and chlamydia) diagnoses among app-using GBM than among nonusers [14]. Counterintuitively, the same meta-analysis found no significant differences in HIV infection rates between app users and nonusers [14], suggesting that there may be some nuance to the association between app use and sexual risk among GBM.

Notably, high rates of some risky sexual behaviors—such as condomless anal intercourse (CAI), group sex, and sexualized drug use—have been reported among app-using GBM [15-19], but reviews have highlighted the need to directly compare users and nonusers [6,14]. A few recent studies have documented a higher likelihood of CAI, group sex, and a greater number of concurrent sexual partnerships among app-using GBM than among nonusers [15,20-24]. However, most studies comparing the risk behaviors of app-using and nonusing GBM have relied on homogenous samples (eg, GBM from Washington, District of Columbia [17]) and are from non-US countries (eg, China [22-24] and Nigeria [21]), making it unclear whether their findings generalize to more diverse samples of GBM from across the United States.

Despite a number of studies showing higher risk behavior in app-using GBM, there are also data suggesting that app-using GBM may also engage in higher levels of *protective* behaviors than nonusers. Several studies have found that app users report being tested for HIV more frequently than their app nonusing counterparts [17,24,25], and one study of GBM receiving HIV and STI screening in San Diego found that GBM who reported using Grindr were more likely to be regular pre-exposure prophylaxis (PrEP) users than those who did not report using Grindr [26]. However, it is unclear whether this finding generalizes to users of GSN apps beyond Grindr, and more studies on the association between GSN app use and PrEP use are needed. Taken together, these findings paint a complex picture, suggesting that although app users have been shown to engage in some risky behaviors more frequently than nonusers, they may also be more likely to engage in risk mitigation and prevention practices, perhaps recognizing their risk of STI infection.

It is important to note that most studies examining GSN app use among GBM recruited samples *directly from dating apps* (eg, [15-19]), which underscores the need for additional research comparing risky sexual behaviors and relevant health outcomes between app-using GBM and their app nonusing counterparts. Furthermore, there is a dearth of research examining differences in risk behaviors among GBM primarily using GSN apps specific to lesbian, gay, bisexual, transgender, and queer/questioning individuals (eg, Grindr) and GBM primarily using sexuality nonspecific apps (eg, Tinder). As sexual partnerships pursued online differ from those pursued through in-person venues [14], it is also possible that risky behaviors may vary by *type* of web-based venue. Supporting the self-selection argument, research suggests that GBM-specific apps are more often used for *hookups* with casual partners than for pursuing long-term relationships [27].

### This Study

In summary, further research is needed to understand how risk profiles differ between app-using and app nonusing GBM, as well as to identify the types of GSN apps that may be associated with higher levels of risk among this population. Thus, this study aimed to address these gaps in the literature by examining sexual risk and protective behaviors as well as HIV and STI prevalence among a diverse sample of GBM including users of GSN apps specific to GBM, users of sexuality nonspecific apps, and app nonusers. We hypothesized that GBM who use GSN apps would report greater HIV and STI prevalence, as well as higher levels of sexual risk behaviors, compared to GBM who do not use GSN apps. In addition, we hypothesized that GBM who use GSN apps would report greater *protective* behaviors, such as pre-PrEP use and HIV testing, despite or perhaps *because of* heightened levels of sexual risk behavior. Finally, we conducted exploratory analyses to examine whether these outcomes differ between GBM using *only* GBM-specific GSN apps and GBM using *only* sexuality nonspecific GSN apps. Given the increasing popularity of these apps [7,8] as well as the increasing rates of STIs in the United States [4], this is both a necessary and timely avenue of research to pursue.

## Methods

### Recruitment

This study used baseline data from a larger experimental study aimed at understanding the causal impact of discrimination on various health behaviors. Respondents were recruited in November 2020 from the web-based crowdsourcing platform Prolific and were eligible to participate if they (1) identified as gay, bisexual, or queer; (2) identified as cisgender male; and (3) were US residents. Prolific is similar to Amazon’s Mechanical Turk; however, it is geared toward academic research, allows niche recruitment, and offers higher data quality [28]. Eligible respondents were invited to complete an anonymous survey assessing their engagement in various health behaviors. Respondents provided informed consent before completing the survey and were compensated US $2.70 for their time and effort. On average, the survey took 18.88 minutes to complete.

### Ethics Approval

The study protocol was reviewed and approved by the University of Colorado Boulder Institutional Review Board (protocol 20-0441) and was conducted in accordance with the Declaration of Helsinki.

### Study Measures

Respondents completed an investigator-developed quantitative survey that included a series of structured questionnaires assessing demographics, GSN app use, and sexual behaviors (refer to Multimedia Appendix 1 for complete survey measures).

### Demographic Characteristics

Respondents were asked demographic questions, including their age, race, ethnicity, highest educational attainment, individual income, geographic location, and relationship status.

### GSN App Use

Respondents were asked whether they used online dating apps, and if so, to indicate which apps they currently have profiles or accounts on (choosing all that applied). Response options included Grindr, Tinder, Scruff, Bumble, Hinge, GROWLr, Jack’d, Hornet, and other. Participants who selected *Other* were asked to list any additional apps on which they had profiles or accounts. The average weekly activity was assessed with the following item: “Please estimate the number of hours per week you spend on online dating apps such as the ones listed above.”

### Motivation for GSN App Use

Motivation for GSN app use was assessed using a measure developed by Goedel and Duncan [29]. Respondents who endorsed using GSN apps were asked, “Which best describes your reason for using these apps?” Response options included, “I want to ‘kill time’ when bored,” “I want to make friends with other gay and bisexual men,” “I want to meet other gay and bisexual men to date,” “I want to find a boyfriend or other romantic partner,” and “I want to meet other gay and bisexual men to have sex with.”

### Number of Sexual Partners

Respondents were asked, ‘In the past year, with how many partners have you had anal sex?’ After examining the response distribution, and consistent with prior studies [24,30], this variable was dichotomized before analysis to create a binary partner number variable (0=less than 3 sexual partners, 1=3 or more sexual partners).

### Condomless Anal Intercourse

Past-year CAI was assessed with a single item asking respondents, “In the past year, with how many partners have you had unprotected anal sex?” After examining the response distribution, and again consistent with prior studies [22,24,30], this variable was dichotomized before analysis to create a binary CAI variable (0=have not had CAI in the past year, 1=have had CAI in the past year).

### Sexualized Drug Use

Respondents were asked to indicate how frequently they consumed alcohol and cannabis before having sex by responding to the following two items: “In the past year, how much of the time did you drink alcohol before you had sexual intercourse?” and “In the past year, how much of the time did you smoke marijuana before you had sexual intercourse?” The response options for each item ranged from 1 (never) to 5 (always). In addition, respondents were given a list of 6 drugs commonly associated with *chemsex* (eg, ecstasy, poppers, and gamma-hydroxybutyrate) [31] and were asked to report whether they had ever used each drug before engaging in sexual intercourse. Items were summed to create a single score for recreational drug use before sex, ranging from 0 to 6.

### Previous STI Diagnosis

Respondents were asked if they had ever been diagnosed with an STI other than HIV (0=have not been diagnosed, 1=have been diagnosed), and if so, which STI. Response options included chlamydia, genital herpes, gonorrhea, hepatitis, human papillomavirus, syphilis, trichomoniasis, and other. Participants who selected *Other* were asked to list any additional STIs they had previously been diagnosed with.

### HIV Testing and Serostatus

Respondents were asked whether they had ever been tested for HIV (0=no, 1=yes), and if so, what their HIV status was (0=HIV negative, 1=HIV positive).

### PrEP Use

PrEP use was assessed using a single item asking respondents, “PrEP is short for pre-exposure prophylaxis. It is a medication that HIV-negative individuals can take to prevent HIV. Do you use PrEP?” (0=do not use PrEP, 1=do use PrEP).

### Sexual Sensation Seeking

Sexual sensation seeking was assessed using an abbreviated version of the Sexual Sensation Seeking scale [32], which assesses an individual’s tendency to seek novel or risky sexual simulation. Respondents were asked to respond to 6 items (eg, “I like new and exciting sexual experiences and sensation”) on a scale ranging from 1 (not at all like me) to 4 (very much like me). Items were averaged to create a single score ranging from 1 to 4, with higher scores indicating greater sexual sensation seeking (*α*=.78).

### Statistical Analysis

All analyses were conducted using R (version 4.0.3). To compare GSN app users with nonusers on continuous outcomes of interest (eg, sexualized drug use), we ran a series of linear regressions with user status as the independent variable (nonuser=−0.5, GSN user=0.5). To compare GSN users with nonusers on categorical outcomes of interest (eg, past-year CAI), we ran a series of logistic regressions with user status as the independent variable. All regression analyses comparing GSN users with nonusers included relationship status (in a relationship=−0.5, not in a relationship=0.5) as a covariate, as relationship status differed significantly between the 2 groups (*P*<.001). Among GSN users, we conducted bivariate correlation tests to examine associations between hours spent per week on GSN apps and continuous outcomes of interest and point-biserial correlation tests to examine associations between hours spent per week on GSN apps and categorical outcomes of interest.

Finally, to examine the differential associations between behavior and GBM-specific versus sexuality nonspecific GSN apps, we conducted a series of exploratory analyses to investigate whether sexual risk behavior and substance use differed between those using *only* GBM-specific GSN apps (ie, Grindr, Scruff, GROWLr, Jack’d, and Hornet) and those using *only* sexuality nonspecific GSN apps (ie, Tinder, Bumble, and Hinge). Respondents who reported using both GBM-specific and sexuality nonspecific GSN apps (n=39) were excluded from the analyses. To compare exclusive users of GBM-specific apps with exclusive users of sexuality nonspecific apps on categorical outcomes of interest, we ran a series of logistic regressions with app preference (sexuality nonspecific only=−0.5, GBM-specific only=0.5) as the independent variable. To compare these users on the continuous outcomes of interest, we ran a series of 2-tailed independent samples *t* tests.

## Results

### Sample Characteristics

In total, 230 individuals completed the web-based survey. A total of 7 respondents self-reported their sexual orientation as heterosexual and were thus excluded. Data were examined for invalid survey response patterns (eg, failed attention checks, invariability in responses, and speeding), and no additional respondents were identified for exclusion. Thus, the final sample consisted of 223 respondents. The respondents were aged, on average, 31.90 (SD 10.06; range 21-78) years. The sample mostly consisted of non-Hispanic White participants (155/223, 69.5%) and included respondents from 40 states and the District of Columbia. GSN users were less likely to report being in a relationship (*P*<.001) and more likely to be a racial minority (*P*=.003) than nonusers. No other significant differences in demographics emerged between GSN users and nonusers (Table 1).

Nearly half (104/223, 47%) of the participants reported using GSN apps. Among GSN users, the most common reason for using these apps was to *kill time* when bored (43/104, 41.3%), followed by meeting other gay and bisexual men to have sex with (23/104, 22.1%), making friends with other gay and bisexual men (19/104, 18.3%), meeting other gay and bisexual men to date (10/104, 9.6%), and wanting to meet a boyfriend or other romantic partner (9/104, 8.7%). Respondents reported spending an average of 4.88 (SD 8.08) hours per week on these apps. The most common apps GSN users endorsed were Grindr (68/104, 65.4%), followed by Tinder (60/104, 57.7%), Scruff (27/104, 26.0%), Bumble (15/104, 14.4%), Hinge (14/104, 13.5%), GROWLr (11/104, 10.6%), Jack’d (8/104, 8.0%), and Hornet (4/104, 3.8%). Of 104 GSN users, 40 (38.5%) reported using 2 or more apps.

**Table 1:** Demographics overall and by user group.

Variable				Overall (N=223)		App users (n=104)		Nonusers (n=119)		*P* value	
Age (years), mean (SD)				31.90 (10.06)		31.12 (8.53)		32.59 (11.22)		.28	
**Sexual orientation, n (%)**											.99
	Gay		100 (44.8)		47 (45.2)		53 (44.5)				
	Bisexual		104 (46.6)		48 (46.2)		56 (47.1)				
	Other		19 (8.5)		9 (8.7)		10 (8.4)				
**Relationship status, n (%)**											*<.001* ^a^
	Single		122 (54.7)		70 (67.3)		52 (43.7)				
	Partnered (monogamous)		91 (40.8)		27 (26.0)		64 (53.8)				
	Partnered (nonmonogamous)		10 (4.5)		7 (6.7)		3 (2.5)				
**Race, n (%)**											*.003*
	White		159 (71.3)		65 (62.5)		94 (79.0)				
	Black or African American		21 (9.4)		13 (12.5)		8 (6.7)				
	Asian		17 (7.6)		14 (13.5)		3 (2.5)				
	American Indian or Alaska Native		1 (0.4)		1 (1.0)		0 (0.0)				
	Native Hawaiian or other Pacific Islander		0 (0.0)		0 (0.0)		0 (0.0)				
	Two or more races		7 (3.1)		1 (1.0)		6 (5.0)				
	**Hispanic or Latinx—any race**									.74	
		Hispanic or Latino	22 (9.9)		11 (10.6)		11 (9.2)				
		Not Hispanic or Latino	201 (90.1)		93 (89.4)		108 (90.8)				
**Education, n (%)**											.63
	Less than high school		2 (0.9)		0 (0.0)		2 (1.7)				
	High school or general educational development		22 (9.9)		9 (8.7)		13 (10.9)				
	Some college		63 (28.3)		29 (2.8)		34 (28.6)				
	Associate degree or technical certification		18 (8.1)		10 (9.6)		8 (67.2)				
	Bachelor’s degree		84 (37.7)		41 (39.4)		43 (36.1)				
	Master’s degree		28 (12.6)		11 (10.6)		17 (14.3)				
	Doctoral or professional degree		6 (2.7)		4 (3.8)		2 (1.7)				
**Annual household income, n (%)**											.35
	< US $25,000		60 (26.9)		21 (20.2)		39 (32.8)				
	US $25,000-$49,999		65 (29.1)		35 (33.7)		30 (25.2)				
	US $50,000-$74,999		47 (21.1)		23 (22.1)		24 (20.2)				
	US $75,000-$99,999		24 (10.8)		13 (12.5)		11 (9.2)				
	US $100,000-$149,999		19 (8.5)		9 (8.7)		10 (8.4)				
	> US $150,000		8 (3.6)		3 (2.9)		5 (4.2)				
**Location of residence, n (%)**											.46
	Rural		24 (11.8)		10 (9.6)		14 (11.8)				
	Suburban		110 (49.3)		48 (46.2)		62 (52.1)				
	Urban		89 (39.9)		46 (44.2)		43 (36.1)				

^a^Significant *P* values are italicized.

### GSN App Use, HIV and STI Prevalence, and Sexual Behavior

The zero-order correlations between sexual risk and protective behaviors are presented in Table 2.

Descriptive statistics for sexual behavior overall and by user group, as well as results of the regression analyses, are presented in Tables 3 and 4. Controlling for relationship status, GSN users reported higher levels of sexual sensation seeking than nonusers. GSN users also reported more frequent use of recreational drugs before sex, alcohol use before sex, and cannabis use before sex compared with nonusers. GSN users were more than 5 times more likely to report past-year CAI, 8 times more likely to report 3 or more sexual partners in the past year, and 3 times more likely to report a past STI diagnosis than nonusers. GSN users were also more likely than nonusers to report having ever taken an HIV test and using PrEP. HIV seropositivity rates were not significantly different between GSN users and nonusers.

Among GSN users (n=105), hours spent per week on GSN apps were positively associated with sexual sensation seeking (*r*=0.28; *P*=.003); frequency of recreational drug use before sex (*r*=0.25; *P*=.01); and the likelihood of past-year CAI (*r*=0.37; *P*<.001), 3 or more sexual partners in the previous year (*r*=0.38; *P*<.001), a past STI diagnosis (*r*=0.31; *P*=.001), HIV testing (*r*=0.26; *P*=.01), and PrEP use (*r*=0.44; *P*<.001). Hours spent on GSN apps were not associated with alcohol use before sex, cannabis use before sex, or HIV serostatus (*Ps*>.47).

**Table 2:** Zero-order correlations between sexual risk and protective behaviors.

Variable		1	2	3	4	5	6	7	8	9
**Past-year condomless anal intercourse**										
	*r*	—^a^	—	—	—	—	—	—	—	—
	*P* value	—	—	—	—	—	—	—	—	—
**Three or more sexual partners in the past year**										
	*r*	0.35	—	—	—	—	—	—	—	—
	*P* value	<.001	—	—	—	—	—	—	—	—
**Previous sexually transmitted infection diagnosis**										
	*r*	0.31	0.25	—	—	—	—	—	—	—
	*P* value	<.001	<.001	—	—	—	—	—	—	—
**Ever been tested for HIV**										
	*r*	0.41	0.25	0.29	—	—	—	—	—	—
	*P* value	<.001	<.001	<.001	—	—	—	—	—	—
**HIV+ serostatus**										
	*r*	0.13	0.06	0.39	0.14	—	—	—	—	—
	*P* value	.28	.76	<.001	<.05	—	—	—	—	—
**Pre-exposure prophylaxis use**										
	*r*	0.26	0.48	0.34	0.22	0.01	—	—	—	—
	*P* value	<.001	<.001	<.001	<.001	.79	—	—	—	—
**Alcohol use before sex**										
	*r*	0.28	0.18	0.07	0.22	−0.08	0.12	—	—	—
	*P* value	<.001	<.01	.31	<.001	.10	.08	—	—	—
**Cannabis use before sex**										
	*r*	0.18	0.15	0.13	0.20	0.02	0.13	0.39	—	—
	*P* value	<.01	<.05	.05	<.01	.94	.06	<.001	—	—
**Recreational drug use before sex**										
	*r*	0.35	0.30	0.38	0.26	0.41	0.17	0.16	0.21	—
	*P* value	<.001	<.001	<.001	<.001	<.001	<.05	<.05	<.01	—
**Sexual sensation seeking**										
	*r*	0.55	0.29	0.37	0.33	0.21	0.19	0.33	0.28	0.44
	*P* value	<.001	<.001	<.001	<.001	<.01	<.01	<.001	<.001	<.001

^a^Not applicable.

**Table 3:** Logistic regression analyses of geosocial networking app users versus nonusers.

Variable^a^	Overall (N=223)	App users (n=104)	Nonusers (n=119)	Adjusted odds ratio (95% CI)	*P* value
Past-year condomless anal intercourse, n (%)	103 (46.2)	64 (61.5)	39 (32.8)	5.46 (2.60-11.48)	*<.001* ^b^
Three or more sexual partners in the past year, n (%)	53 (23.8)	44 (42.3)	9 (7.6)	8.26 (3.71-18.40)	*<.001*
Previous sexually transmitted infection diagnosis, n (%)	44 (19.7)	30 (28.8)	14 (11.8)	3.40 (1.62-7.11)	*.001*
Ever been tested for HIV, n (%)	145 (65.0)	75 (72.1)	70 (58.8)	3.00 (1.58-5.69)	*<.001*
HIV+serostatus, n (%)	8 (3.6)	6 (5.8)	2 (1.7)	1.73 (0.31-9.55)	.53
Pre-exposure prophylaxis use, n (%)	24 (10.8)	17 (16.3)	7 (5.9)	2.90 (1.12-7.56)	*.03*

^a^All regression models comparing GSN users and nonusers controlled for participant relationship status.

^b^Significant *P* values are italicized.

**Table 4:** Linear regression analyses of geosocial networking app users versus nonusers.

Variable^a^	Overall (N=223)	App users (n=104)	Nonusers (n=119)	Coefficient (*B*)	*t* test (*df*)	*P* value
Alcohol use before sex, mean (SD)	1.90 (1.02)	2.16 (1.06)	1.67 (0.93)	0.62	4.59 (220)	*<.001* ^b^
Cannabis use before sex, mean (SD)	1.52 (0.87)	1.65 (0.97)	1.40 (0.75)	0.34	2.84 (220)	*.01*
Recreational drug use before sex, mean (SD)	0.65 (1.16)	0.90 (1.39)	0.43 (0.87)	0.53	3.30 (220)	*.001*
Sexual sensation seeking, mean (SD)	2.19 (0.70)	2.41 (0.68)	1.99 (0.65)	0.52	5.75 (220)	*<.001*

^a^All regression models comparing GSN users and nonusers controlled for participant relationship status.

^b^Significant *P* values are italicized.

### GBM-Specific Versus Sexuality Nonspecific GSN Apps

Among GSN users, 38 respondents reported using *only* GBM-specific GSN apps (ie, Grindr, Scruff, GROWLr, Jack’d, and Hornet), whereas 27 respondents reported using *only* sexuality nonspecific GSN apps (ie, Tinder, Bumble, and Hinge). GSN users who only endorsed using only GBM-specific GSN apps spent more hours per week on GSN apps than GSN users who only endorsed using sexuality nonspecific GSN apps (t_63_=2.34; *P*=.02). The most common reason for using GBM-specific apps was to meet other gay and bisexual men to have sex with (17/38, 45%), whereas the most common reason for using sexuality nonspecific apps was to kill time when bored (14/27, 52%).

Exclusive users of GBM-specific apps reported higher levels of sexual sensation seeking than exclusive users of sexuality nonspecific apps (t_128_=5.14; *P*<.001). Exclusive users of GBM-specific apps also reported more frequent recreational drug use before sex (t_63_=2.54; *P*=.01) and were more likely to report past-year CAI (OR 16.10, 95% CI 4.46-58.14; *P*<.001), 3 or more sexual partners in the previous year (OR 4.89, 95% CI 1.53-15.61; *P*=.004), a previous STI diagnosis (OR 26.00, 95% CI 3.20-211.49; *P*=.002), and HIV testing (OR 6.13, 95% CI 1.83-20.47; *P*=.003). Alcohol use before sex, cannabis use before sex, PrEP use, and HIV rates were similar between both the groups (*P*>.11).

## Discussion

### Principal Findings

As GBM continue to bear a disproportionate burden of the HIV and STI epidemic [1], it is important to examine how contextual factors, such as the proliferation of mobile dating apps, may shape STI risk within this community. This study suggests that GSN app use—dichotomous user versus nonuser status and time spent on apps among users—is associated with higher rates of sexual risk in GBM across a range of outcome measures. Conversely, the findings also suggest that GSN app use is associated with increased odds of engaging in health *protective* behaviors. Furthermore, among GSN users, we found that exclusive users of GBM-specific apps (eg, Grindr), as opposed to sexuality nonspecific apps (eg, Tinder), reported greater sexual risk taking despite similar rates of PrEP use.

Recent reviews have called for more studies to compare health risk and protective behaviors between GSN users and nonusers, as much of the extant literature consists of GBM samples recruited directly from GSN apps, which inherently limits findings to the prevalence of sexual risk among GSN users [6,14]. In our study that directly compared GSN using GBM with GSN nonusing GBM, we found that although GSN app use was associated with increased odds of a previous STI diagnosis, past-year CAI, and 3 or more sexual partners in the preceding year, it was also related to greater engagement in STI risk reduction strategies (ie, PrEP use and HIV testing). This is encouraging, as it suggests that users may recognize the inherent risks associated with sexual behavior and actively engage with strategies for risk mitigation and prevention. Furthermore, our finding that GSN app use was associated with greater levels of sexualized drug use contributes to the emerging literature on this topic [15]. As there are well-documented event-level associations between substance use, sexual risk taking, and subsequent STI and HIV infection [33-35], this association—and the mechanisms behind it—warrants further exploration. For instance, GSN app use may reflect greater integration with GBM communities where sexualized drug use is normative [12,13]. Interventions and policy strategies aimed at reducing this practice among GBM should integrate both HIV and substance use education [12,13], and GSN apps could serve as a unique platform for disseminating these harm reduction initiatives.

In addition to comparing GSN users with nonusers, we provide a more nuanced exploration of the risks associated with GBM-specific (vs sexuality nonspecific) app use, which has been overlooked in the previous literature. Our findings indicate that men who used only GBM-specific apps (vs sexuality nonspecific apps) were more likely to report past-year CAI, 3 or more sexual partners in the preceding year, a previous STI diagnosis, and more frequent recreational drug use before sex, suggesting that the use of GBM-specific apps is associated with a higher risk user profile than sexuality nonspecific apps. The higher rates of risk behavior associated with GBM-specific apps may be driven by differences in motives for app use (eg, sexual partner seeking among GBM-specific app users vs killing time for users of sexuality nonspecific apps). This aligns with prior evidence suggesting that GBM-specific apps (eg, Grindr and Jack’d) are used primarily for *hookups* rather than dating [14]. Interestingly, despite greater levels of risk among GBM using only GBM-specific apps, the rates of PrEP use did not significantly differ between the groups; however, PrEP uptake was still low across both groups (13%). It is important to note that advertisements for PrEP are more common on GBM-specific dating sites, suggesting that further evaluation of the efficacy of such advertisements would be informative. Interestingly, although the rates of PrEP use were similar among app users, men who used only GBM-specific apps were more likely to report HIV testing than men who used only sexuality nonspecific apps. This may be due, in part, to the fact that GBM-specific apps encourage users to report their HIV serostatus as well as the date of their last HIV test.

### Limitations

Despite the novel contributions made to the existing literature, this study has several limitations. Among GSN users, we were unable to determine whether self-reported risk behaviors occurred with partners met through GSN apps. Future studies should examine risk behaviors with partners who met online compared with those who met offline. Our self-report measures of sexual risk and protective behaviors also lacked nuance. For instance, although CAI is a risk factor for STIs, our measure may not accurately reflect respondents’ personal levels of HIV risk, as it did not assess whether respondents who engaged in CAI were having sex with a seroconcordant partner, were regularly taking PrEP at the time of intercourse, or had an undetectable viral load (if HIV positive). Furthermore, our measure of PrEP use was a single yes-no question; however, evidence suggests that daily or almost-daily (4 or more pills per week) adherence to PrEP is necessary to maintain its efficacy [36]. In addition, this study’s data regarding protective behaviors were limited to dichotomous measures of lifetime prevalence of STI and HIV testing and diagnoses, which did not align with the time frames for measures of app use and sexual risk behavior. Future studies should use more detailed self-report measures of risk and protective behaviors, such as the Timeline Follow-Back [37,38]. In addition, although our sample was diverse in terms of age (21-78 years) and geographic location (40 US states and the District of Columbia; urban, suburban, and rural areas), participants were predominantly White, whereas GBM of color—particularly Latino and Black GBM—are at far higher risk of HIV and other STI than White GBM [39]. Participants were also recruited as a convenience sample, which may limit the generalizability of our findings. Finally, this study was cross-sectional in nature. As such, we are unable to determine whether the relationships reported are causal, and if so, the directionality of these effects. For instance, although it is possible that GSN app use may *causally* influence the sexual risk behavior of GBM, it is also possible that GBM with a greater propensity for risk taking may self-select into these web-based environments. The fact that GSN app users reported higher levels of sexual sensation seeking lends credence to the self-selection hypothesis. Future prospective studies should examine how risk levels change in response to the initiation of GSN app use. Regardless of whether GSN app use causally influences risk taking, the fact remains that risk behaviors are elevated among GBM using these apps. Thus, these apps represent an important venue for targeted public health communications aimed at reducing STI risk.

### Conclusions

To reduce disparities in STI and HIV infection and transmission rates, it is critical for researchers, clinicians, and public health officials to maintain an up-to-date awareness of contextual determinants of STI risk that are specific to high-risk populations. The findings of our study suggest that GSN apps, which have played an important role in reducing barriers and facilitating partner seeking among sexual minorities in the 10 years since their inception [9,10], may also be a useful pathway for evaluating and reducing STI risk in GBM. At the individual level, clinicians should ask about the use of dating apps when discussing patient history, given the relationship between the use of GSN apps and risky sexual behaviors. On a larger scale, GSN apps may be a useful tool for public health messaging and STI risk reduction interventions that address individual- and societal-level factors driving sexual risk among this population. The use of Grindr as a platform for PrEP advertisements in recent years is a promising start, but outreach efforts should be increased to reach GBM who prefer to use other GBM-specific apps (eg, Jack’d) or sexuality nonspecific apps (eg, Tinder), and further research is needed to evaluate the efficacy of such efforts.

## Appendix

Multimedia Appendix 1 Web-based survey measures.

## Abbreviations

CAI: condomless anal intercourse
GBM: gay, bisexual, and other men who have sex with men
GSN: geosocial networking
LGB: lesbian, gay, and bisexual
MSM: men who have sex with men
PrEP: pre-exposure prophylaxis
STI: sexually transmitted infection

## References

[1] (2020). Diagnoses of HIV infection in the United States and dependent areas, 2018. Centers for Disease Control and Prevention.

[2] (2020). Sexual transmission and viral hepatitis. Centers for Disease Control and Prevention.

[3] (2021). Centers for Disease Control and Prevention.

[4] (2021). Sexually Transmitted Disease Surveillance 2019. Centers for Disease Control and Prevention.

[5] (2012). Substance Abuse and Mental Health Services Administration.

[6] Zou H, Fan S (2017). Characteristics of men who have sex with men who use smartphone geosocial networking applications and implications for HIV interventions: a systematic review and meta-analysis. Arch Sex Behav.

[7] Curry D (2022). Dating App Revenue and Usage Statistics (2021). Business of Apps.

[8] Meisenzahl M (2020). These charts from Match Group show more people are turning to online dating during the pandemic. Business Insider.

[9] Brown A (2020). Lesbian, gay and bisexual online daters report positive experiences – but also harassment. Pew Research Center.

[10] Jaspal R (2017). Gay men’s construction and management of identity on Grindr. Sex Cult.

[11] Liau A, Millett G, Marks G (2006). Meta-analytic examination of online sex-seeking and sexual risk behavior among men who have sex with men. Sex Transm Dis.

[12] Chan AS, Tang PM (2021). Application of novel psychoactive substances: Chemsex and HIV/AIDS policies among men who have sex with men in Hong Kong. Front Psychiatry.

[13] Kong TS, Laidler KJ (2020). The paradox for chem-fun and gay men: a neoliberal analysis of drugs and HIV/AIDS policies in Hong Kong. J Psychoactive Drugs.

[14] Wang H, Zhang L, Zhou Y, Wang K, Zhang X, Wu J, Wang G (2018). The use of geosocial networking smartphone applications and the risk of sexually transmitted infections among men who have sex with men: a systematic review and meta-analysis. BMC Public Health.

[15] Goedel WC, Duncan DT (2016). Contextual factors in geosocial-networking smartphone application use and engagement in condomless anal intercourse among gay, bisexual, and other men who have sex with men who use Grindr. Sex Health.

[16] Obarska K, Szymczak K, Lewczuk K, Gola M (2020). Threats to mental health facilitated by dating applications use among men having sex with men. Front Psychiatry.

[17] Phillips G, Grov C, Mustanski B (2015). Engagement in group sex among geosocial networking mobile application-using men who have sex with men. Sex Health.

[18] Landovitz RJ, Tseng CH, Weissman M, Haymer M, Mendenhall B, Rogers K, Veniegas R, Gorbach PM, Reback CJ, Shoptaw S (2013). Epidemiology, sexual risk behavior, and HIV prevention practices of men who have sex with men using GRINDR in Los Angeles, California. J Urban Health.

[19] Rice E, Holloway I, Winetrobe H, Rhoades H, Barman-Adhikari A, Gibbs J, Carranza A, Dent D, Bunlap S (2012). Sex risk among young men who have sex with men who use Grindr, a smartphone geosocial networking application. J AIDS Clinic Res.

[20] Lehmiller JJ, Ioerger M (2014). Social networking smartphone applications and sexual health outcomes among men who have sex with men. PLoS One.

[21] Ogunbajo A, Lodge 2nd W, Restar AJ, Oginni OA, Iwuagwu S, Williams R, Biello K, Mimiaga MJ (2021). Correlates of geosocial networking applications (GSN Apps) usage among gay, bisexual, and other men who have sex with men in Nigeria, Africa. Arch Sex Behav.

[22] Tang W, Best J, Zhang Y, Liu FY, Tso LS, Huang S, Yang B, Wei C, Tucker JD (2016). Gay mobile apps and the evolving virtual risk environment: a cross-sectional online survey among men who have sex with men in China. Sex Transm Infect.

[23] Wang H, Zhang J, Chu Z, Hu Q, Dong W, Huang X, Chen Y, Wang H, He X, Zhang L, Hu Z, Bao R, Li S, Li H, Cui S, Jin X, Ding H, Geng W, Jiang Y, Xu J, Shang H (2020). Risk-taking behaviors and adherence to HIV pre-exposure prophylaxis in users of geosocial networking apps: real-world, multicenter study. J Med Internet Res.

[24] Xu J, Yu H, Tang W, Leuba SI, Zhang J, Mao X, Wang H, Geng W, Jiang Y, Shang H (2018). The effect of using geosocial networking apps on the HIV incidence rate among men who have sex with men: eighteen-month prospective cohort study in Shenyang, China. J Med Internet Res.

[25] Bien CH, Best JM, Muessig KE, Wei C, Han L, Tucker JD (2015). Gay apps for seeking sex partners in China: implications for MSM sexual health. AIDS Behav.

[26] Hoenigl M, Little SJ, Grelotti D, Skaathun B, Wagner GA, Weibel N, Stockman JK, Smith DM (2020). Grindr users take more risks, but are more open to Human Immunodeficiency Virus (HIV) pre-exposure prophylaxis: could this dating app provide a platform for HIV prevention outreach?. Clin Infect Dis.

[27] Macapagal K, Kraus A, Moskowitz DA, Birnholtz J (2020). Geosocial networking application use, characteristics of app-met sexual partners, and sexual behavior among sexual and gender minority adolescents assigned male at birth. J Sex Res.

[28] Palan S, Schitter C (2018). Prolific.ac—a subject pool for online experiments. J Behav Exp Finance.

[29] Goedel WC, Duncan DT (2015). Geosocial-networking app usage patterns of gay, bisexual, and other men who have sex with men: survey among users of Grindr, a mobile dating app. JMIR Public Health Surveill.

[30] Balaji AB, Bowles KE, Hess KL, Smith JC, Paz-Bailey G, NHBS study group (2017). Association between enacted stigma and HIV-related risk behavior among MSM, National HIV Behavioral Surveillance System, 2011. AIDS Behav.

[31] Bourne A, Reid D, Hickson F, Torres-Rueda S, Weatherburn P (2015). Illicit drug use in sexual settings ('chemsex') and HIV/STI transmission risk behaviour among gay men in South London: findings from a qualitative study. Sex Transm Infect.

[32] Kalichman SC, Rompa D (1995). Sexual sensation seeking and Sexual Compulsivity Scales: reliability, validity, and predicting HIV risk behavior. J Pers Assess.

[33] Heath J, Lanoye A, Maisto SA (2012). The role of alcohol and substance use in risky sexual behavior among older men who have sex with men: a review and critique of the current literature. AIDS Behav.

[34] Mustanski B (2008). Moderating effects of age on the alcohol and sexual risk taking association: an online daily diary study of men who have sex with men. AIDS Behav.

[35] Ritchwood TD, DeCoster J, Metzger IW, Bolland JM, Danielson CK (2016). Does it really matter which drug you choose? An examination of the influence of type of drug on type of risky sexual behavior. Addict Behav.

[36] Spinner CD, Boesecke C, Zink A, Jessen H, Stellbrink HJ, Rockstroh JK, Esser S (2016). HIV pre-exposure prophylaxis (PrEP): a review of current knowledge of oral systemic HIV PrEP in humans. Infection.

[37] Pachankis JE, McConocha EM, Reynolds JS, Winston R, Adeyinka O, Harkness A, Burton CL, Behari K, Sullivan TJ, Eldahan AI, Esserman DA, Hatzenbuehler ML, Safren SA (2019). Project ESTEEM protocol: a randomized controlled trial of an LGBTQ-affirmative treatment for young adult sexual minority men's mental and sexual health. BMC Public Health.

[38] Weinhardt LS, Carey MP, Maisto SA, Carey KB, Cohen MM, Wickramasinghe SM (1998). Reliability of the timeline follow-back sexual behavior interview. Ann Behav Med.

[39] (2021). HIV and gay and bisexual men. Centers for Disease Control and Prevention.

