# Supplementary material for: Geosocial Networking App Use Associated With Sexual Risk Behavior and Pre-exposure Prophylaxis Use Among Gay, Bisexual, and Other Men Who Have Sex With Men: Cross-sectional Web-Based Survey
Source: JMIR Form Res. 2022 Jun 13;6(6):e35548. doi: 10.2196/35548 (PMC9237762; doi:10.2196/35548)
Supplement: Multimedia Appendix 1 [file formative_v6i6e35548_app1.docx]

**MULTIMEDIA APPENDIX**

Survey Measures

How old are you (years)?

________________________________________________________________

What is your race or ethnicity? Select all that apply.

- White or Caucasian
- African American or Black
- Hispanic or Latino
- American Indian or Alaskan Native
- Asian
- Native Hawaiian or Pacific Islander
- Other: ________________________________________________

Which of the following best describes you?

- Heterosexual (straight)
- Gay
- Queer
- Bisexual
- Uncertain
- Other ________________________________________________

Please indicate the highest level of education you have completed.

- High school diploma or GED
- Some college
- Associate’s degree or Technical Certification
- Bachelor’s degree
- Master’s degree
- Doctoral (PhD) or Professional degree (MD, JD, etc.)
- Less than high school

What is your best estimate of YOUR total annual household income? If you are not sure, please give your best guess.

- Less than $25,000 per year
- $25,000 to $49,999 per year
- $50,000 to to $74,999 per year
- $75,000 to $99,000 per year
- $100,000 to $149,000 per year
- $150,000 and up per year

Which of the following best describes your relationship status?

- I am single
- I am partnered (monogamous)
- I am partnered (non-monogamous)

In which state do you currently reside?

▼ Alabama ... I do not reside in the United States

How would you describe the community where you currently live?

- Urban
- Suburban
- Rural

In the past year, with how many partners have you had anal sex?

________________________________________________________________

Display This Question:

If If In the past year, with how many partners have you had anal sex? Text Response Is Greater Than 0

In the past year, with how many partners have you had **unprotected** anal sex?

________________________________________________________________

Have you ever been tested for HIV?

- Yes
- No

Display This Question:

If Have you ever been tested for HIV? = Yes

What is your HIV status?

- HIV-positive
- HIV-negative

PrEP is short for pre-exposure prophylaxis. It is a medication that HIV-negative individuals can take to prevent HIV. Do you use PrEP?

- Yes
- No

Have you ever been diagnosed with a sexually transmitted disease or infection?

- Yes
- No

Display This Question:

If Have you ever been diagnosed with a sexually transmitted disease or infection? = Yes

Which of the following have you been diagnosed with?

- Chlamydia
- Gonorrhea
- Genital Herpes
- Hepatitis
- Human Papillomavirus (HPV)
- Syphilis
- Trichomoniasis
- Other: ________________________________________________

Have you ever used any of the following drugs before engaging in sexual intercourse?

|  | Yes | No |
| --- | --- | --- |
| Ecstasy |  |  |
| Methamphetamine |  |  |
| Erectile dysfunction drugs (e.g., Viagra) |  |  |
| Gamma-hydroxybutyrate (GHB) |  |  |
| Amyl Nitrite ("Poppers") |  |  |
| Mephedrone |  |  |

In the past year, how much of the time did you drink alcohol before you had sexual intercourse?

- Never
- Almost never
- Sometimes
- Almost always
- Always

In the past year, how much of the time did you smoke marijuana before you had sexual intercourse?

- Never
- Almost never
- Sometimes
- Almost always
- Always

Please indicate which geosocial-networking/online dating apps you currently have profiles or accounts on [select all that apply].

- Grindr
- Scruff
- Jack'd
- Tinder
- Hornet
- Bumble
- GROWLr
- Hinge
- Other [please specify] ________________________________________________
- Not applicable. I do not use geosocial-networking/online dating apps.

Display This Question:

If Please indicate which geosocial-networking/online dating apps you currently have profiles or acco... != Not applicable. I do not use geosocial-networking/online dating apps. is NOT selected

|  |
| --- |

Please estimate the number of **hours per week** you spend on online dating apps such as the ones listed above.

________________________________________________________________

Display This Question:

If Please indicate which geosocial-networking/online dating apps you currently have profiles or acco... != Not applicable. I do not use geosocial-networking/online dating apps. is NOT selected

Which best describes your reason for using these apps?

- I want to 'kill time' when bored
- I want to make friends with other gay and bisexual men
- I want to meet other gay and bisexual men to date
- I want to find a boyfriend or other romantic partner
- I want to meet other gay and bisexual men to have sex with

Please read the following statements and rate whether or not the statements reflect your own experiences, thoughts or feelings. Indicate the response that best fits your reaction to the statement.

|  | Not at all like me | Somewhat like me | Often like me | Very much like me |
| --- | --- | --- | --- | --- |
| I like the "uninhibited" sexual encounters |  |  |  |  |
| The physical sensations are the most important thing about having sex |  |  |  |  |
| I enjoy the sensation of intercourse without a condom |  |  |  |  |
| My sexual partners probably think I'm a "risk taker" |  |  |  |  |
| When it comes to sex, physical attraction is more important to me than how well I know the person |  |  |  |  |
| I like new and exciting sexual experiences and sensations |  |  |  |  |
